# Health literacy of undergraduate students at a university in the Western Cape: A survey

**DOI:** 10.4102/hsag.v30i0.2902

**Published:** 2025-05-30

**Authors:** Talitha Crowley, Million Bimerew, Thabani Noncungu, Furaha Akimanimpaye, Jeffrey Hoffman, Portia Bimray, Mussie Melesse, Benjamin Kutumbuka, Jennifer A. Chipps

**Affiliations:** 1 School of Nursing, Faculty of Community and Health Sciences, University of the Western Cape, Cape Town, South Africa

**Keywords:** health literacy, healthcare, information, students, university

## Abstract

**Background:**

Health literacy is critical in the lives of young people such as university students to ensure that they can access information about health risks and implement appropriate health promotion and disease prevention strategies.

**Aim:**

This study aimed to investigate the health literacy of undergraduate students by describing the level of health literacy, personal and situational factors influencing health literacy, and health care utilisation.

**Setting:**

The study was conducted at a university in the Western Cape.

**Methods:**

A quantitative descriptive survey was used (*N* = 953). Data were collected using a paper-based questionnaire that included demographic variables, the 47-item European Health Literacy Survey Questionnaire (HLS-EU-Q47) and questions on healthcare utilisation and overall health. Descriptive analysis was conducted.

**Results:**

Over two-thirds of the respondents were classified under ‘problematic health literacy’ (*n* = 372, 39.0%) or ‘inadequate literacy’ (*n* = 274, 28.8%). Only 220 respondents (23.1%) had ‘sufficient’ health literacy, and only 87 (9.1%) achieved an ‘excellent’ rating. Respondents who searched for health information (*p* = 0.006) and accessed healthcare (*p* = 0.014) in the last 6 months had significantly higher levels of health literacy and this was associated with a better overall health rating (*p* < 0.001).

**Conclusion:**

The study highlighted significant gaps in health literacy among university students, particularly in the domains of disease prevention and health promotion, indicating the need for targeted intervention.

**Contribution:**

The study provides useful information on the current health literacy of young adults (university students) that can be used to plan health promotion activities.

## Introduction

Health literacy includes the ‘knowledge, motivation and competencies of persons to access, understand, appraise and apply health information’ in the domains of healthcare, health promotion and disease prevention, with the overall goal of optimal health and well-being throughout the lifespan (Pelikan et al. 2019). The definition is specifically relevant in the modern information society where young people have access to various sources of information and need the competencies to understand, appraise and translate information into health behaviours (Pelikan et al. 2019). An individual’s health literacy influences health behaviours, such as healthcare utilisation, and consequently, health status and illness behaviours (Pelikan et al. 2019).

Insufficient health literacy increases the frequency of healthcare visits or may cause delays in seeking care, whereas good health literacy improves the effective use of healthcare services, lowering healthcare costs (Viktorsson et al. 2019).

Health literacy plays an important role in the lives of young people, such as university students, to ensure that they can access information about health risks and implement appropriate health promotion and disease prevention strategies throughout their lives and the lives of others (Broder et al. 2017, 2020). Various studies on the health literacy of youth and university students have been conducted internationally.

In Sweden, more than a third of youth had low levels of health literacy (Viktorsson et al. 2019). A study in Turkey among university students found that 45% had problematic health literacy levels; gender (being female) and education in the health field were associated with health literacy (Uysal, Ceylan & Koc 2020). An Australian study among university-based medical, allied health or nursing degree programmes found that health literacy profiles differed across student groups (Mullan et al. 2017).

In France and Spain, only 36.5% of health and social care students had sufficient health literacy (Juvinya-Canal et al. 2020). Similarly, in China, students’ health literacy needed improvement (Zhang et al. 2016).

Health literacy can be determined by a number of personal and situational determinants or factors such as gender, age, level of education, socioeconomic status and geographical context (Pelikan et al. 2019). A study among university students in Greece found that economic factors (family income), demographic factors (gender) and health behaviours and/or risks (alcohol use, smoking, physical activity) were associated with health literacy (Vozikis, Drivas & Milioris 2014). However, there is a paucity of literature on the health literacy of university students in the African and South African contexts.

This study thus aims to investigate the health literacy of undergraduate students in a university in the Western Cape by describing the level of health literacy, personal and situational determinants of health literacy, and healthcare utilisation. Information from this study may assist in developing contextually appropriate health literacy programmes.

## Research methods and design

### Setting

The study is conducted at a university in the Western Cape, South Africa, located in a low resource area. The university has around 27 000 students located in seven faculties, each with varied online teaching presence, listed in declining order of size are Economic and Management Sciences; Arts and Humanities; Natural Sciences; Community and Health Sciences; Education; Law; and Dentistry.

### Design

The study used a quantitative survey design with a self-administered questionnaire.

### Population and sampling

The population was all registered students at the time of data collection (~*N* = 27 000). The inclusion criterion was being registered at the university in 2023. Students < 18 years were excluded because obtaining parental consent for participation in research was not possible.

In 2020, the Faculty of Economic and Management Sciences had a headcount of 23.6% or 5608 students, followed, respectively, by the Faculty of Arts and Humanities with a headcount of 4542 (19.1%); Faculty of Natural Sciences with a headcount of 3975 (16.7%); Faculty of Community and Health Sciences’ headcount of 3627 (15.3%); Faculty of Education with 2826 (11.9%); Law with a headcount of 2385 (or 10%); and Dentistry with 767 students (3.2%). Using a 6% error and estimated proportion *p* = 50 % for each faculty, a total of 1688 students were required in the sample. Using a 60% response rate, at least 1013 students were to be included, around 150 students per faculty (Table 1). Achieving proportional sampling within faculties was not feasible because of logistical constraints. Instead, a convenience sampling approach was adopted, where students were invited to participate through in-class recruitment and campus-based data collection points.

**TABLE 1:** Demographic data.

Characteristics	Male (*n* = 338)				Female (*n* = 599)				Total (*n* = 953)†				Test		*P* *
	*n*	%	Mean	s.d.	*n*	%	Mean	s.d.	*n*	%	Mean	s.d.	*U*	*χ* ^2^	
Age (years)	-	-	20.8	2.6	-	-	20.7	2.9	-	-	20.7	2.8	−1.453	-	0.146
**Faculty**															
Arts and Humanities	30	8.9	-	-	42	7.0	-	-	72	7.7	-	-	-	35.07	< 0.001
Community and Health Sciences	47	13.9	-	-	155	25.9	-	-	202	21.6	-	-	-		
Dentistry	24	7.1	-	-	31	5.2	-	-	55	5.9	-	-	-		
Economics and Management Sciences	46	13.6	-	-	48	8.0	-	-	96	10.1	-	-	-		
Education	52	15.4	-	-	111	18.5	-	-	166	17.4	-	-	-		
Law	65	19.2	-	-	132	22.0	-	-	204	21.4	-	-	-		
Natural Sciences	74	21.9	-	-	80	13.4	-	-	158	16.6	-	-	-		
**Year level**															
Junior level	176	52.1	-	-	333	55.6	-	-	516	54.1	-	-	-	1.08	0.299
Senior level	162	47.9	-	-	266	44.4	-	-	437	45.9	-	-	-		

* , Nonbinary excluded from analysis because of small sample size;

† , Sixteen respondents who indicated nonbinary added to the total.

s.d., standard deviation.

### Instrumentation

A self-administered questionnaire, which included the 47-item European Health Literacy Survey Questionnaire (HLS-EU-Q47) scale, was used. The scale measures health literacy and has been used widely in Europe and other countries with good validity and reliability (Pelikan et al. 2019; Sørensen et al. 2013). The instrument also has a short version, HLS-EU-Q16, that has been tested in various settings in Europe (Mekhail et al. 2022; Pedro et al. 2023). The scale measured individual health literacy in three domains: healthcare (16 items), disease prevention (15 items) and health promotion (16 items). Each of these domains is measured in terms of the four steps of information management: gaining access or obtaining information, discriminate between sources of information (understand), appraise and/or judge and personalise health information, appropriately apply and/or use relevant health information. Higher scores reflect higher levels of health literacy. Score calculations are presented under data analysis.

Statements were measured on a Likert scale: very easy = 4, rather easy = 3, rather difficult = 2, very difficult = 1 (Pelikan et al. 2019). The authors added five demographic questions, and six questions related to healthcare utilisation and health status. Cronbach’s alpha was calculated for the health literacy domains in this study: healthcare (alpha = 0.83), disease prevention (alpha = 0.87) and health promotion (alpha = 0.88).

### Data collection

Data were collected using a paper-based questionnaire administered by the research teams who had been trained in data collection and research. Permission was obtained from the university registrar and university faculty deans. Potential respondents were approached on the university campus and informed about the study. Interested respondents were provided with an information sheet and written informed consent document. Following consent, the respondents completed the questionnaires and submitted them to a closed box with a submission slit. The instrument took 10 min on average to complete.

### Data analysis

Data were entered and analysed using Statistical Package for Social Sciences (SPSS), version 29 (IBM, Armonk, New York, United States). Descriptive statistics were used to describe the variables using frequency distribution tables. A total health literacy score and mean domain and subdomain scores were calculated. Higher values suggest higher health literacy. For ease of comparability, the general health literacy score was standardised on a scale from 0 to 50 (index = (mean – 1)*(50/3) as recommended by the developers (Pelikan et al. 2019). Four levels of health literacy have been defined: inadequate health literacy (0–25 pts or 50%), problematic health literacy (> 25–33 pts or 66%), sufficient health literacy (> 33–42 pts or 80%) and excellent health literacy (> 42–50 pts or top 20%) (Pelikan et al. 2019). The year levels of study were re-coded, with Years 1 and 2 classified as junior and Years 3 and 4 as senior. Chi-square, Mann–Whitney (*U*) and Kruskal–Wallis tests were used to test for associations between demographic variables, healthcare utilisation, health status and health literacy using a significance level of *p* < 0.05.

### Ethical considerations

Ethical clearance to conduct this study was obtained from University of the Western Cape Faculty of Community and Health Sciences (reference no.: HS22/8/28) and respondents provided written informed consent. All respondents were assured of confidentiality and anonymity of any information provided. Data are being kept secured and scored according to the university protocols.

## Results

### Demographic details

The survey included 953 undergraduate respondents, predominantly female (*n* = 599, 62.9%). The average age was 20.7 years (± 2.8), with ages ranging from 18 years to 50 years. Most respondents were junior level students, that is, Year 1 or 2 (*n* = 509, 54.3%), with the highest representation from the Faculty of Community and Health Sciences (*n* = 202, 21.6%), followed closely by the Faculty of Law (*n* = 197, 21.0%) (Table 1). The distribution of gender was significantly different across Faculties with the Community and Health Sciences faculty having the highest representation of females (*n* = 155, 25.9%) and the Natural Sciences having the highest male representation (*n* = 74, 21.9%) (χ^2^ = 35.07, *p* ≤ 0.001).

### Healthcare utilisation and overall health

Nearly three quarter of the respondents (*n* = 671, 70.4%) reported that in the last 6 months, they have searched for health information online two or more times, with a significantly higher proportions of female compared to male respondents, (*n* = 218, 64.5% vs. *n* = 439, 73.3%, χ^2^ = 9.04, *p* = 0.011) (Table 2). Google was the most frequently used search platform (714, 86.7%), with females significantly more likely to use it than males (*n* = 469, 88.5% vs. *n* = 232, 83.2%) (χ^2^ = 4.5, *p* = 0.034).

**TABLE 2:** Healthcare utilisation and overall health.

Variables	Male (*n* = 338)		Female (*n* = 599)		Total (*n* = 953)**		*χ*^2^ test	*P* *
	*n*	%	*n*	%	*n*	%		
**Search for health information online in the last 6 months**								
Never	59	17.5	69	11.5	129	13.5	9.040	0.011*
Once	61	18.0	91	15.2	153	16.1		
Two or more times	218	64.5	439	73.3	671	70.4		
**Where did you search for information?†**								
Google	232	83.2	469	88.5	714	86.7	4.500	0.034*
Social media	8	2.9	31	5.8	40	4.9	3.500	0.060
Databases	35	12.5	47	8.9	83	10.1	2.710	0.100
Other	12	4.3	11	2.1	23	2.8	3.280	0.070
**Access to healthcare services in the last 6 months** ‡								
Never	99	29.3	153	25.5	252	26.9	7.900	0.019*
Once	125	37.0	188	31.4	313	33.4		
Two or more times	114	33.7	258	43.1	372	39.7		
**Reason for accessing health care services§**								
Health promotion or disease prevention	55	23.0	157	35.2	214	30.7	10.820	0.001*
Acute illness	59	24.7	106	23.8	168	24.1	0.072	0.789
Emergency	17	7.1	35	7.8	52	7.4	0.120	0.729
Chronic illness	18	7.5	33	7.4	52	7.4	0.004	0.950
**Healthcare services accessed¶**								
Campus clinic	31	13.2	60	13.5	93	13.3	0.031	0.859
Public health clinic	76	31.8	128	28.7	207	29.7	0.715	0.398
Private healthcare, for example, GP	67	28.0	162	36.3	233	33.4	4.805	0.028*
Pharmacy	57	23.8	97	21.7	155	22.2	0.394	0.530
Other	11	4.6	15	3.4	29	4.2	0.654	0.419
**Healthcare provider††**								
Doctor	108	45.2	231	51.8	346	49.6	2.710	0.099
Nurse	64	26.8	130	29.1	196	28.1	0.430	0.512
Pharmacist	56	23.4	78	17.5	135	19.3	3.492	0.062
Traditional healer	1	0.4	1	0.2	2	0.3	0.202	0.653
Natural medicine practitioner	3	1.3	1	0.2	4	0.6	2.849	0.091
Other	10	4.2	20	4.5	33	4.7	0.033	0.855
**Overall health rating‡‡**								
Below average (poor, fair)	38	11.2	104	17.4	146	15.3	6.293	0.012*
Above average health (good, very good, excellent)	300	88.8	495	82.6	807	84.7		

* , Significant at *p* < 0.05;

** , Sixteen respondents who indicated nonbinary added to the total but excluded from gender analysis due to small sample size;

† , Male; *n* = 279; female; *n* = 530; total; *n* = 824;

‡ , Male; *n* = 338; female; *n* = 599; total; *n* = 953;

§ , Male; *n* = 239; female; *n* = 446; total; *n* = 698);

¶ , Male; *n* = 239; female; *n* = 446; total; *n* = 698;

†† , Male; *n* = 239; female; *n* = 446; total; *n* = 698;

‡‡ , Male; *n* = 338; female; *n* = 599; total; *N* = 953.

Just over a quarter of respondents (*n* = 252, 26.9%) reported that they did not access healthcare services in the last 6 months. A significantly higher proportion of females (*n* = 258, 43.1%) accessed health services two or more times in the last 6 months compared to males (*n* = 114, 33.7%) (χ^2^ = 7.9, *p* = 0.019). Among those who accessed health services (*n* = 698), nearly a third sought services for health promotion or disease prevention (*n* = 214, 30.7%), with most opting for private healthcare (*n* = 233, 33.4%) or a public health clinic (*n* = 207, 29.7%). A significantly higher proportion of females accessed health promotion services compared to males (*n* = 157, 35.2% vs. *n* = 55, 23.0%) (χ^2^ = 10.82, *p* = 0.001) and visited a general practitioner compared to males (*n* = 162, 36.3% vs. *n* = 67, 28.0%) (χ^2^ = 4.805, *p* = 0.028). Just under half of respondents reported visiting a medical doctor in the last 6 months (*n* = 346, 49.6%).

Despite most (*n* = 807, 84.7%) of the respondents rating their health as above average, a significantly lower proportion of females (*n* = 495, 82.6%) did so compared to males (*n* = 300, 88.8%) (χ^2^ = 6.293, *p* = 0.012). When considering the nonbinary gender category, four respondents (25%) rated their health as below average, which is significantly lower compared to the other gender categories (*χ*^2^ = 7.413, *p* = 0.025) (not shown in Table 2).

### General health literacy index

**FIGURE 1:**
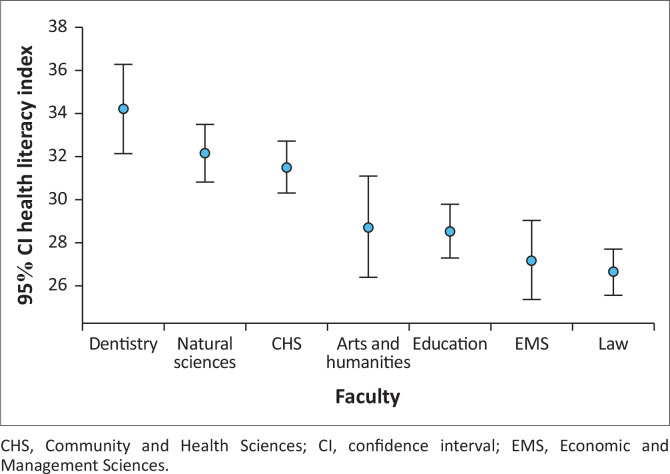
Health literacy index across categories of faculty.

The respondents had an average Health Literacy Index of 29.6 ± 8.7/50, ranging from 0 to 50. The overall Health Literacy Index score differed significantly across categories faculties (*K* = 68.979, *p* < 0.001), with the highest median scores reported in Dentistry and Natural Sciences (34.1 ± 7.8, *n* = 55 and 32.2 ± 8.6, *n* = 158), respectively, and the lowest in the Law and Economic and Management Sciences Faculties (26.6 ± 7.8, *n* = 204 and 27.2 ± 9.1, *n* = 96; Figure 1). There were no significant differences between the scores of junior and senior students (*p* = 0.622), although senior students had higher scores compared to junior students (131 ± 24.2 vs. 129.8 ± 25.1). The correlation between age and the Health Literacy Index was significant, but weak (*r* = 0.096, *p* = 0.003).

Respondents who searched for health information in the last 6 months (29.9 ± 8.7 vs. 27.4 ± 8.9, *U* = 2.726; *p* = 0.006) had significantly higher Health Literacy Index scores compared to those who did not. Similarly, those who accessed health services in the last 6 months had significantly higher Health Literacy Index scores compared to those who did not (29.9 ± 8.7 vs. 28.5 ± 8.7; *U* = 2.455, *p* = 0.014). Furthermore, respondents who rated their health as above average had significantly higher Health Literacy Index scores compared to those who rated their health as below average (30.1.9 ± 8.7 vs. 26.9 ± 8.6; *U* = 4.424, *p* < 0.001).

Most of the respondents’ Health Literacy Index score was classified as ‘Problematic health literacy’ (*n* = 372, 39.0%), followed by ‘Inadequate literacy’ (*n* = 274, 28.8%), with only 220 (23.1%) classified as having ‘Sufficient’ health literacy and 87 (9.1%), achieved an ‘Excellent’ score. More male respondents (*n* = 150, 44.5%) were classified as having ‘Problematic Health Literacy’, in contrast to 216 (36.1%) female respondents (Table 3), with the differences approaching significance.

**TABLE 3:** General health literacy index.

Health literacy level	Male (*n* = 338)		Female (*n* = 599)		Total (*n* = 953)†		*χ* ^2^	*P*
	*n*	%	*n*	%	*n*	%		
Inadequate health literacy (0% – 25%)	93	27.5	178	29.7	274	28.8	6.721	0.081
Problematic health literacy (> 25% – 33%)	150	44.4	216	36.1	372	39.0		
Sufficient health literacy (> 33% – 42%)	69	20.4	148	24.7	220	23.1		
Excellent health literacy (> 42% – 50%)	26	7.7	57	9.5	87	9.1		

† , Sixteen respondents who indicated nonbinary added to the total and nonbinary excluded from analysis because of small sample size.

### Health literacy domains

**FIGURE 2:**
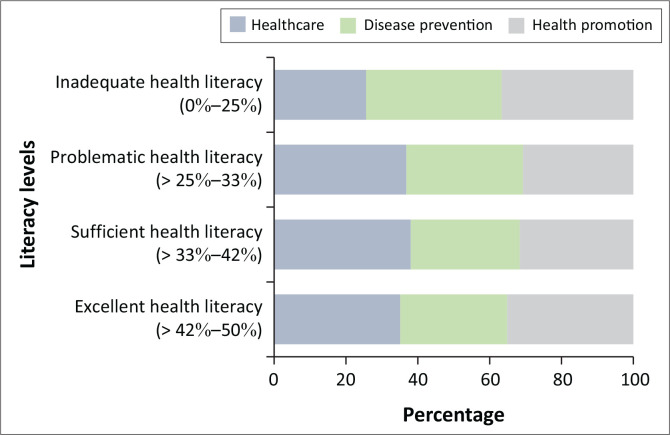
Domains of health literacy.

When considering the health literacy domains, over two-thirds of the respondents (*n* = 633, 66.4%) fell in the inadequate and/or problematic health literacy category in the disease prevention domain, followed by the health promotion domain (*n* = 607, 63.7%) and healthcare (*n* = 559, 58.7%; Table 4 and Figure 2).

**TABLE 4:** Domains of health literacy (*N* = 953).

Health literacy level	Health care		Disease prevention		Health promotion	
	*n*	%	*n*	%	*n*	%
Inadequate health literacy (0% – 25%)	235	24.7	347	36.4	336	35.3
Problematic health literacy (> 25% – 33%)	324	34.0	286	30.0	271	28.4
Sufficient health literacy (> 33% – 42%)	282	29.6	225	23.6	234	24.6
Excellent health literacy (> 42% – 50%)	112	11.8	95	10.0	112	11.8

### Information management

The study assessed four information management steps in the three domains, namely, access, understand, appraise and apply. Overall, the healthcare domain had the highest health information management mean (2.88 ± 0.520) with the ‘apply’ activity having the highest mean (3.11 ± 0.584). Disease prevention had the lowest health information management mean (2.69 ± 0.635) with the ‘appraise’ activity having the lowest mean (2.43 ± 0.782). In the health promotion domain, access had the lowest mean (2.64 ± 0.742) (Table 5).

**TABLE 5:** Information management.

Domains	Access		Understand		Appraise		Apply		Total	
	Mean	s.d.	Mean	s.d.	Mean	s.d.	Mean	s.d.	Mean	s.d.
Healthcare	3.01	± 0.679	2.92	± 0.634	2.79	± 0.589	3.11	± 0.584	2.88	± 0.520
Disease prevention	2.85	± 0.781	3.00	± 0.810	2.43	± 0.782	2.62	± 0.795	2.69	± 0.635
Health promotion	2.64	± 0.742	2.81	± 0.682	2.76	± 0.864	2.81	± 0.819	2.75	± 0.625

When comparing the domain means across categories of gender, respondents in the nonbinary category had the highest means in all the domains, although not significant. When comparing the differences between males and females, female respondents had a significantly higher mean in the disease prevention domain (2.72 ± 0.648 vs. 2.64 ± 0.601; *U* = 2.060, *p* = 0.039) (Table 6).

**TABLE 6:** Healthcare literacy domain mean score per gender.

Domains	Male (*n* = 338)		Female (*n* = 599)		Nonbinary (*n* = 16)		All (*n* = 953)		Mann–Whitney U*	*P*
	*n*	Mean	*n*	Mean	*n*	Mean	*n*	Mean		
Total healthcare	2.87	± 0.477	2.88	± 0.541	3.07	± 0.567	2.88	± 0.520	0.293	0.769
Total disease prevention	2.64	± 0.601	2.72	± 0.648	2.76	± 0.778	2.69	± 0.635	2.060	0.039
Total health promotion	2.76	± 0.592	2.73	± 0.645	3.04	± 0.482	2.75	± 0.625	−0.792	0.429

* , Nonbinary excluded from analysis because of small sample size.

## Discussion

The study aimed to investigate the health literacy of undergraduate students in a university in the Western Cape by describing the level of health literacy, personal and situational determinants of health literacy and health care utilisation.

When considering the Health Literacy Index score, more than two-thirds (67.8%) of respondents had problematic (39.0%) or inadequate literacy (28.8%). Only 23.1% of respondents had sufficient health literacy and 9.1% had excellent health literacy. This is consistent with studies amongst students in Turkey where 45% of students had problematic health literacy (Uysal et al. 2020). In France and Spain, 43.4% and 20.1% of students in nursing, social work, primary education and special education, respectively, exhibited inadequate or problematic health literacy, as assessed by the short version of the HLS-EU-Q47, HLS-EU-Q16 (Juvinya-Canal et al. 2020).

Similarly, 37% of young adults in Sweden answering the HLS-EU-Q16 had insufficient and/or problematic health literacy (Viktorsson et al. 2019). A systematic review of 21 cross-sectional studies on university students’ health literacy found that the level of health literacy among university students seems to be insufficient and needs to be improved (Kühn et al. 2022).

When comparing the domains, it is evident that the healthcare domain scores are generally higher than those for disease prevention and health promotion. This disparity suggests that respondents may feel more comfortable navigating health care services than engaging with preventive and promotional health strategies. The lower scores in the disease prevention domain, particularly in appraisal and application, highlight a critical gap that could impact individuals’ ability to effectively prevent diseases.

Personal and situational determinants of health literacy that were investigated in this study included gender, age and the faculty the students were enrolled at. There were observable differences in health literacy levels between genders, with a higher proportion of females classified as having sufficient health literacy compared to males. However, these differences were not significant (*p* = 0.081). In France and Spain, females had higher levels of health literacy, but the study did not find significance (Juvinya-Canal et al. 2020; Kühn et al. 2022). Inconsistent findings were also reported in a systematic review where four studies measured higher levels of health literacy among females and two measured higher health literacy among males (Kühn et al. 2022). When considering the health literacy domains, females had significantly higher mean scores in the disease prevention domain (*p* = 0.039). This may be because female students are likely to access health care services for sexual reproductive health (Juvinya-Canal et al. 2020; Maricic et al. 2021).

There was no difference between the health literacy levels when comparing junior (i.e. first and second year) and senior (third and fourth year) students. Further, the bivariate correlation between age and health literacy was weak, albeit significant. A study in France and Spain also reported no significant association between age and health literacy (Juvinya-Canal et al. 2020). A systematic review of university students’ health literacy found a positive association with age in most studies, indicating that older students may have an advanced ability to navigate the healthcare system and engage with healthcare professionals (Kühn et al. 2022).

Health literacy varied significantly across faculties (*K* = 68.979, *p* < 0.001), with the highest scores observed in Dentistry and Natural Sciences, and the lowest in Law and Economic and Management Sciences. This disparity is likely influenced by curricular exposure to health-related content, as students in health and science-based disciplines may develop stronger skills in understanding and applying health information. In contrast, faculties with less emphasis on health topics may not provide the same opportunities to build health literacy. These findings underscore the role of academic background in shaping health literacy and suggest the need for interdisciplinary approaches to strengthen health literacy across all faculties. This was similar to a study among health professional students in Australia, where medical students had the highest health literacy scores (Mullan et al. 2017). Studies in Australia and Europe also found that health literacy differs across student groups or degrees (Juvinya-Canal et al. 2020; Mullan et al. 2017), with students in Education in France and Spain were less likely to have sufficient health literacy compared to nursing students (Juvinya-Canal et al. 2020).

This study indicates that the majority of respondents (82.5%) searched for health information online at least twice in the past 6 months, with the majority using Google for their searches (86.7%). Google may serve as a low-cost and easily accessible source for searching for health information compared to using social media and databases. However, it may not yield accurate health information. A systematic review by Kuhn et al. (2022) found that although the Internet is the most popular method for students to access information, it is also associated with the poorest health literacy scores. A quarter of the respondents (26.9%) did not access healthcare services and those who did primarily sought care for acute illnesses. Females were more likely to search for health information and access health care services and female and nonbinary respondents were more likely to rate their health as below average compared to males.

Respondents who searched for health information in the last 6 months and those who accessed health care services had significantly higher levels of health literacy which was associated with an above-average health rating. The findings concur with Mullan et al. (2017) that health literacy is associated with health care utilisation and overall wellbeing. A study in Sweden, though not specifically of university students, but of young adults seeking healthcare services, found that ‘insufficient’ or ‘problematic’ health literacy was linked to lower reliance on the healthcare system, an increased likelihood of seeking treatment for mental health symptoms, greater health anxiety and reduced trust in the healthcare system (Viktorsson et al. 2019).

### Implications and recommendations

The study highlights the need for targeted health literacy interventions, particularly in disease prevention and health promotion, through curriculum integration and workshops. Improving access to health resources and services can help students manage their health and enhance academic success. Given the reliance on Google for health information, universities should teach students how to critically assess online sources. Tailored interventions for nonhealth faculties and gender-sensitive strategies for male students are also essential. Finally, embedding health literacy into student support services and institutional policies will equip students to better manage their health, supporting both academic and personal success.

### Strengths and limitations

A strength of the study is that to the best of our knowledge, this is the first study to report health literacy amongst university students in South Africa. Limitations of the study relate to the fact that it was conducted in one university and due to sampling constraints, an equal proportion of respondents in the various faculties could not be attained. The final sample did not strictly reflect faculty population sizes. Weighting was not applied to adjust for these differences, meaning faculties with smaller enrolments may be overrepresented relative to their actual student population. This limits the generalisability of the findings.

## Conclusion

The results of this study highlight significant gaps in health literacy among university students, particularly in the domains of disease prevention and health promotion, highlighting the need for health literacy interventions that are specifically tailored to different domains of health literacy. Strategies should aim to improve individuals’ capabilities to evaluate and use health information, especially concerning disease prevention and health promotion. Although university students generally feel confident in accessing healthcare, there are notable deficiencies in their understanding and application of health information in these areas. Bridging these gaps with targeted interventions is essential for improving overall health literacy and enabling individuals to make well-informed health decisions. Moreover, expanding access to resources and support systems can further empower individuals to seek and effectively use health information, ultimately leading to improved health outcomes and better academic success.
